# State-of-the-art considerations in small cell lung cancer brain metastases

**DOI:** 10.18632/oncotarget.19333

**Published:** 2017-07-18

**Authors:** Rimas V. Lukas, Vinai Gondi, David O. Kamson, Priya Kumthekar, Ravi Salgia

**Affiliations:** ^1^ Department of Neurology, Northwestern University, Chicago, IL, USA; ^2^ Department of Radiation Oncology, Northwestern Medicine Cancer Center Warrenville, Northwestern Medicine Chicago Proton Center, Northwestern University, Warrenville, IL, USA; ^3^ Department of Neurology, University of Chicago, Chicago, IL, USA; ^4^ Department of Medical Oncology and Therapeutics, City of Hope, Duarte, CA, USA

**Keywords:** brain metastases, chemotherapy, pathophysiology, radiation therapy, small cell lung cancer

## Abstract

**Background:**

Small cell lung cancer (SCLC) frequently leads to development of brain metastases. These unfortunately continue to be associated with short survival. Substantial advances have been made in our understanding of the underlying biology of disease. This understanding on the background of previously evaluated and currently utilized therapeutic treatments can help guide the next steps in investigations into this disease with the potential to influence future treatments.

**Design:**

A comprehensive review of the literature covering epidemiology, pathophysiology, imaging characteristics, prognosis, and therapeutic management of SCLC brain metastases was performed.

**Results:**

SCLC brain metastases continue to have a poor prognosis. Both unique aspects of SCLC brain metastases as well as features seen more universally across other solid tumor brain metastases are discussed. Systemic therapeutic studies and radiotherapeutic approaches are reviewed.

**Conclusions:**

A clearer understanding of SCLC brain metastases will help lay the framework for studies which will hopefully translate into meaningful therapeutic options for these patients.

## INTRODUCTION

Important advances in our understanding of brain metastases are underway [1, 2]. While there have been many recent reviews of brain metastases [3–6] as well as more focused reviews on non-small cell lung cancer (NSCLC) brain metastases [7–9] there has been a paucity of contemporary reviews focusing on small cell lung cancer (SCLC) brain metastases. A broad overview of SCLC can be found in a recent review [10]. Within this manuscript we will narrow the focus exclusively to SCLC brain metastases. A comprehensive understanding of this cancer’s involvement of the brain will set the stage for the next steps in optimizing its management. A number of patients succumb to SCLC in the brain and we have to define better biology and therapeutics.

### Epidemiology

Lung cancer metastases to the brain affect more patients than any other solid tumor metastases in the U.S. Due to the higher incidence of non-small cell lung cancer (NSCLC) when compared to SCLC, it comprises a higher percentage of patients with brain metastases. However, SCLC appears to have a higher propensity for the central nervous system (CNS). In patients with non-metastatic lung cancer the risk of brain metastases in SCLC appears to be double that of NSCLC. Factors which influence this risk are being clarified. The incidence of SCLC brain metastases does not appear to be influenced by race. It does appear to be higher, however, in younger patients (< 60) compared to older [11]. It is unclear if it is influenced by gender, with at least one study noting a significantly higher incidence of development of metachronous brain metastases and a shorter brain metastases free interval in men with limited stage disease. [12] Increasing pathologic stage correlates with higher incidence of brain metastases. Therapeutic management, such as the extent of resection (complete vs. incomplete) of the primary tumor influences this as well [13]. Like all solid tumors, SCLC has the potential to spread to the leptomeninges. The 2 year cumulative incidence of leptomeningeal involvement in SCLC patients is ∼10%. Many, but not all, of these patients also have concomitant brain metastases [14]. An older large retrospective study from 1969 to 1980 by the National Cancer Institute demonstrated leptomeningeal involvement in 25% of SCLC patients at 3 years. Leptomeningeal involvement most often occurred in the context of extra-CNS relapse of disease. The greatest risk factors for leptomeningeal involvement appear to be other CNS involvement at diagnosis as well as metastases to other distant sites such as liver and bone [15]. These findings, however, may not reflect contemporary incidence rates.

### Prognosis

Prognosis for patients with SCLC remains poor. Earlier SCLC-specific analyses, of patients from trials conducted from 1983 to 2005, using the Radiation Therapy Oncology Group (RTOG) recursive partitioning analysis (RPA) classification system revealed a median overall survival (OS) of 4.9 months (range 0.3–40.3 months) in patients with newly diagnosed SCLC brain metastases. Patients with older age, poorer performance status, uncontrolled primary tumor, and/or extracranial metastases had worse outcomes, correlating with the RTOG RPA classes seen across the aggregated histologies of all solid tumor brain metastases [16]. In the more contemporary disease-specific graded prognostic assessment (GPA) system the same prognostic factors remained valid. Additionally, the number of brain metastases influenced outcomes. Whether this is secondary to the biology of oligo- vs. multi-metastatic disease, the imaging eras (CT vs MRI) when the studies were conducted, the treatments utilized in the management of the two conditions, or a combination of these factors is unknown. Median OS for all patients with SCLC brain metastases was seen to be 4.90 months [17] however ranged from 3.0–14.8 depending on these four validated risk factors (KPS, age, number of brain metastases, and extracranial metastases). Further support is lent from smaller studies focused on SCLC brain metastases where performance status, presence of extracranial metastases, and the number of brain metastases have all been associated with OS [18]. Survival in recurrent SCLC is presumably poorer than in the newly diagnosed setting. There is less data to clearly define prognosis in that specific setting, however. In patients with leptomeningeal involvement, survival is limited with OS reported as 1.3–2.4 months [14]. While improvements in survival have been seen across a range of solid tumor histologies with brain metastases, this has unfortunately not been the case in SCLC.

### Pathophysiology

SCLC is a tumor which arises from pulmonary neuroendocrine cells as well as other potential candidate cells such as alveolar type 2 cells [10]. It has the potential to metastasize early and extensively. The brain is a common site for its metastases. This is likely due to the appropriate “seeds” arriving and thriving in an optimal “soil” [19]. Our understanding of this phenomenon has grown since Piaget’s description. It is still limited by a number of factors, including the infrequent biopsy or resection of SCLC brain metastases when compared to other histologies and the limited number of autopsies performed on cancer patients in the modern era. It is clear that in other solid tumors there is genetic divergence between the primary site and the brain metastases which has been termed “branched evolution” [20]. It is uncertain at this time if this holds true for SCLC brain metastases but has been seen in multiple other primary cancers. We will first review the neuro-anatomic features of SCLC brain metastases before delving into the genomic and proteomic features.

#### Neuro-anatomic localization

SCLC is more likely to be associated with multiple brain metastases as opposed to single brain metastases. Mapping the distribution of SCLC in the brain may further expand our understanding of the interaction between metastatic cells with their micro-environment. It will also help optimize anatomically targeted treatment modalities such as radiation therapy [21]. Our initial understanding of the neuroanatomic localization of SCLC brain metastases arose from autopsy studies. One early single center review of 15,000 autopsies performed between 1969 and 1984 concluded that SCLC metastases were equally distributed throughout the brain in contrast to other lung cancer subtypes that favor posterior circulation territories [22]. This study was limited by the slice thickness of 1cm provided by routine autopsies, rendering its resolution similar to early CT imaging studies and admittedly overlooking smaller brain metastases. Utilizing MRI much smaller metastases can be delineated and the population data can be aggregated into anatomic frequency maps [21, 23, 24]. Using this technique, SCLC was found to favor the cerebellum [21, 24]. This disproportionate distribution raises both pathophysiologic questions regarding the potential trophic factors leading to increased aggregation and likely more importantly, factors leading to facilitated growth in these locations as well therapeutic questions regarding differential radiation dosing. When investigating the pattern of growth, both well-demarcated lesions and diffusely infiltrating ones have been seen on autopsy studies. The vascular co-option which is well described in melanoma brain metastases has not been noted in association with SCLC [25].

In addition to the brain, SCLC can also involve other CNS structures. These patterns of spread are less well studied. Spinal cord parenchyma can be a sight of metastasis. It is relatively rare in comparison to brain metastases, occurring in only ∼2% of SCLC patients [26]. This appears to occur predominantly in the setting of parenchymal brain metastases [14]. Leptomeningeal spread of SCLC can occur metachronously, synchronously, or independent of the diagnosis of brain metastases. When associated with brain metastases, this is somewhat more frequent with posterior fossa metastases [14]. Leptomeningeal involvement can also lead to direct invasion of both the spinal cord and brain parenchyma [15].

#### Genomic and gene-expression profile

Studies of human surgical tissue from SCLC brain metastases have demonstrated significant upregulation and downregulation of genes (Table 1). These genes can be broadly categorized as related to angiogenesis, cell-cell adhesion, immune activity, and survival/proliferation/differentiation pathways. A number of angiogenesis related genes including ANGPT4, PDGFRB, COL4A2, and VEGFA are all upregulated. Some such as ANGPT4 and PDGFRB appear to be uniquely upregulated (50-fold) in SCLC brain metastases when compared to NSCLC brain metastases. Others are profoundly upregulated across numerous histologies pointing towards a potentially more universal role in brain metastases [27]. The nature of these potential roles is yet to be fully defined. Their differential dysregulation between tumor types, however, points towards a histology-specific angiogenic profile within brain metastases. In rat models of SCLC brain metastases inhibition of the sulfonylurea receptor 1 (SUR1) with glyburide has led to decreased intracranial vascular permeability and improvement in cerebral edema [28]. These findings may represent potential avenues for therapeutic investigation.

**Table 1:** Genomic and proteomic abnormalities in SCLC brain metastases

Gene or protein	Function	In SCLC brain metastases
TGFβ1	Immunosuppression, ECM interactions, apoptosis	downregulated
ANGPT4	angiogenic	upregulated
PDGFRB	angiogenic	upregulated
IFNβ1	cytokine, immunosuppresive	upregulated
CXCL10	cytokine, increased anti-tumor activity	upregulated
CEACAM1	cell adhesion	upregulated
PECAM1	cell adhesion	upregulated
KIT	receptor tyrosine kinase, survival/proliferation/differentiation	upregulated
COL4A2	collagen subunit in angiogenesis	upregulated
COL15A1	collagen subunit	upregulated
HSPG2	basement membrane, cell growth	upregulated
TNF	cytotoxic, inflammation	upregulated
VEGFA	angiogenic	upregulated
CD44	cell-cell-ECM adhesion	upregulated
CDH1	cell-cell adhesion	loss of heterozygosity
CX3CR1	adhesion	increased expression
PD-1	immunosupression	limited expression in TILs
PD-L1	immunosuppresion	Expression in ¼ of TILs and TAMs and some tumor cells
SUR1	vascular permeability	Blockade leads to decreased cerebral edema

SCLC, small cell lung cancer; ECM, extra-cellular matrix; TILs, tumor infiltrating lymphocytes; TAMs, tumor associated macrophages.

Cell-cell and cell-extracellular matrix adhesion also appears to be important in SCLC brain metastases with numerous upregulated genes including CEACAM1, PECAM1, HSPG2 and CD44 all upregulated [24]. Additional aberrancies including loss of heterozygosity of the E-cadherin gene CDH1 have been noted as well [29]. Analyses of post-mortem tissue from untreated patients demonstrate SCLC brain metastases have a higher incidence (83%) of expression of the chemokine receptor CX3CR1 when compared to metastases to other organs such as liver (28%), locoregional lymph nodes (14%), and adrenal glands (0%) [30]. Normal lung neuroepithelial cells exhibit an inherent ability to traverse from one location to another via a process dubbed slithering [31]. This can lead to postulation regarding the predisposition of SCLC for early metastasis as well as which chemokines and adhesions molecules play a critical role in SCLC metastasis to the brain. One chemokine of interest, CXCR4, is expressed in SCLC and may play an important role in the mechanism of metastasis to the brain. This is since the brain contains a rich resource of the CXCR4 ligand CXCL12 (also referred to as stromal derived growth factor) [32, 33].

As with many other malignancies, the role of tumor, microenvironment, and the immune system holds substantial interest and has sparked hope for potential therapeutic targets. The underpinnings are still incompletely understood. SCLC brain metastases have been associated with a high amount of astroglial reactivity when compared to NSCLC adenocarcinomas and squamous cell brain metastases [34]. It has been demonstrated in mouse models of adenocarcinoma that tumor cell-astrocyte gap junctions composed of connexin 43 serve as conduits for transfer of cGAMP into astrocytes leading to astrocytic production of IFNα and TNFα. These paracrine signals activate growth and chemoresistance signals [35]. It is uncertain if this also hold true in SCLC. Downregulation of the immunosuppresive TGFβ1 gene, but upregulation of the immunosuppressive IFNβ1 gene have been described in SCLC brain metastases. Upregulation of the pro-inflammatory TNF gene has also been noted [26]. It is difficult to parcel out what is cause and what is consequence. The majority (93.8%) of SCLC brain metastases specimens have demonstrated the presence of tumor-infiltrating lymphocytes (TIL). All of the TIL+ samples have shown CD3+ TILs and the majority have also exhibited CD8+ TILs. CD45RO+ memory TILs were seen in about half of the brain metastases. Their presence was associated with improved median OS (11 months vs. 5 months, *p* = 0.007). Programmed death-1 (PD-1) expression has been seen in only a limited (3.1%) subset of TILs in SCLC brain metastases. However, programmed death ligand-1 (PD-L1) has been detected in a higher percentage of TILs (25%) and tumor infiltrating macrophages (28.1%). PD-L1 expression was also seen in > 5% of tumor cells in over 1/3 of the brain metastases samples [36]. In extra-CNS SCLC increased PD-1 and PD-L1 expression in SCLC tumor cells, possibly mediated via deregulation of KIT and DNA methyltransferase 1 (DNMT1), correlated with cisplatin resistance. It is hypothesized that chronic platinum exposure leads to the upregulation of PD1/PD-L1 in the tumor cells [37]. It is uncertain whether this has an impact on brain metastases. The large number of genetic alterations in SCLC [38] may make it a particularly attractive target for immunotherapies.

SCLC can also arise in the context of previous targeted therapies for non-small cell lung cancers. Sequist, et al. initially described the “transformation” of EGFR mutated adenocarcinomas of the lung upon treatment with tyrosine kinase inhibitors. There was a propensity for these SCLC to also metastasize. It is possible that there is clonal selection with aggressive phenotype of metastasis [39].

### Radiology

#### CT and MRI

Clinical management of SCLC brain metastases begins with their symptomatic or incidental detection with imaging. The clinical role of oncologic imaging is to identify, prognosticate, assist treatment plan formulation, and monitor disease status. Conventionally used techniques yield such information by detecting changes in anatomy. The 3rd edition of the American College of Chest Physicians’ Guidelines for SCLC Diagnosis and Treatment recommends contrast-enhanced brain CT or MRI as the mainstay for staging SCLC without differentiating between the two modalities [40, 41]. In contrast, the National Comprehensive Cancer Network 2017 Clinical Practice Guidelines express preference for MRI over CT for staging in the brain [42]. Generally, its higher resolution makes MRI superior to CT. According to an early study comparing CT vs. MRI for detection of SCLC BMs, CT missed 85% of metastases in the posterior fossa and 97% of all metastases smaller than 5 mm [43]. Similar conclusions were reached comparing the prevalence of brain metastases in patients with newly diagnosed SCLC in the “CT era” (roughly 1980 to 1991) versus the “MRI era” (1991 studied until 2004) [44, 45]. Whereas, the MRI detected prevalence was 24% with almost half of these consisting of asymptomatic patients, CT detected only 10% with a bias for symptomatic lesions. These differences in sensitivity need to be taken into account when evaluating outcome data. For example, the number of brain metastases (single vs. multiple) is a prognostic factor when detected via the more sensitive MRI but is not prognostic when utilizing CT which may under-detect metastases creating the false impression of a single metastasis [44].

While the above-mentioned routine techniques visualize tissue structure, advanced imaging techniques intend to assess tissue properties, such as water or blood flow, or metabolism. Diffusion-weighted imaging of the brain, more specifically apparent diffusion coefficient (ADC) mapping has been well studied in SCLC. Low ADC values are generally associated with high cellularity and anaplasticity in tumors, features closely associated with SCLC brain metastases [46, 47]. While not all studies could distinguish primary tumor types based on MRI ADC values [48], SCLC appears to have the lowest mean and median ADC differentiating it from melanoma, breast cancer, and NSCLC [49]. There may also be a higher intrapatient ADC variability in SCLC brain metastases compared to NSCLC. A comprehensive analysis by Zakaria et al. corroborated the low ADC in SCLC brain metastases, although this feature was shared with melanoma in their analysis [47]. Perfusion-weighted MRI (PWI) and MR spectroscopy (MRS), useful techniques for monitoring progression and aiding differentiation of radiation necrosis vs. tumor recurrence have not been investigated in depth in SCLC.

#### Nuclear imaging

Positron emission tomography (PET) uses radiotracers to visualize metabolic activity *in vivo*. The 2-deoxy-2[^18^F]fluoro-D-glucose (FDG) is the only PET radiotracer at the present that is widely used in a clinical setting and is one of the mainstays for the extracranial staging of SCLC [40, 41]. However, FDG-PET can only detect 45% of SCLC brain metastases seen on MRI or even CT, and is incapable of revealing most lesions not seen on these standard modalities [50, 51]. More so than the low spatial resolution, the cause of this low sensitivity seen with FDG-PET is high physiologic glucose metabolism in the brain masking metastases that may be hypometabolic relative to the cortex or basal ganglia. This is particularly unfavorable for SCLC where 2 out of 3 brain metastases may go undetected, whereas this proportion is only 20% in NSCLC [52].

Other radiotracers have also been evaluated for assessment of brain metastases. One such tracer is radioactive octreotide, intended to harness the neuroendocrine properties of SCLC. While some brain metastases not evident on MRI were detected via the octreotide scans, its low sensitivity did not validate it as a clinical tracer [53, 54]. Mono amino acid radiotracers such as L-[^11^C]methyl-methionine (MET), [^18^F]fluoroethyl-tyrosine (FET), [^18^F]fluoro-L-dopa (FDOPA) and α-[^11^C]methyl-L-tryptophan (AMT) may provide superior contrast to FDG given their lower physiologic uptake in the brain. They have proven useful in the imaging of primary brain tumors [55–57]. Two MET-PET studies with a substantial proportion of lung cancer (> 70% in each) demonstrated the ability to distinguish recurrent metastatic brain tumors from radiation necrosis with a sensitivity and specificity of 75% or above, yet included only two SCLC brain metastases whereas the other did not even specify such [58, 59]. Finally, utilizing kinetic uptake analysis on AMT-PET, a normalized net tryptophan transport could distinguish brain metastases from lung versus breast cancer with an accuracy of 88%, whereas tracer accumulation enabled distinction from glioblastoma with 81% accuracy. However, low sample size did not allow testing of differences between NSCLC and SCLC here either [60].

### Surgery

Due to the frequent multi-metastatic picture in SCLC surgery does not typically have a role in the management of this disease. In the three randomized trials evaluating the role of surgical resection of single brain metastases patients with SCLC were excluded, limiting our understanding of the role of surgical management in this patient population [61–63]. To our knowledge there are no studies specifically evaluating the role of craniotomy and surgical resection of SCLC brain metastases. Due to the propensity of multiple brain metastases and the relative radiosensitivity of these tumors the likelihood of a comprehensive study evaluating surgical resection of SCLC brain metastases is unlikely in the foreseeable future.

### Radiotherapy

Because brain metastases are a frequent problem in patients with SCLC [64], and the burden of brain metastases can impact on quality and length of survival, several prospective trials have examined the use of prophylactic cranial irradiation (PCI) in SCLC patients who present without brain metastases. This is performed for limited disease and the most recent data for extensive disease does not favor PCI. These trials have consistently shown that PCI leads to a reduction in the incidence of brain metastases and a prolongation in survival in patients with limited disease with initial response [65, 66]. In spite of the compelling evidence supporting the use of PCI, a recent study demonstrated that 40% of SCLC patients for whom a survival benefit from PCI has been shown, do not receive PCI due to concerns of cognitive toxicity on the part of both patient and physician [67]. A recent trial of PCI for limited-stage SCLC demonstrated a 62% (95% confidence interval 50–74%) rate of cognitive toxicity [68]. In another study, PCI was associated with a higher rate of decline in patient-reported cognitive function [69]. Observations of cognitive toxicity from PCI for SCLC appear similar to those seen after PCI for locally advanced non-small cell lung cancer and after whole-brain radiotherapy for brain metastases [70–72], and demonstrate a differential sensitivity of memory-related cognitive domains to cranial irradiation. Building upon extensive preclinical and clinical data supporting the memory-specificity and radiosensitivity of the hippocampal neural stem cell compartment [73, 74], an ongoing phase III trial (NRG CC003) seeks to determine whether conformal avoidance of the hippocampal dentate gyrus using intensity-modulated radiotherapy during PCI can prevent cognitive toxicity, while still providing the intracranial control benefit of PCI [75].

In SCLC patients who present with brain metastases but have never been treated with PCI, therapeutic whole-brain radiotherapy is the standard of care. Similar to absent role for surgery in SCLC brain metastases, given their multi-metastatic propensity, radiosurgery is contraindicated for PCI-naïve SCLC patients presenting with brain metastases. However, radiosurgery can be an effective therapeutic option for recurrent or progressive brain metastases after prior PCI or WBRT and can help obviate the need as associated neurotoxicity of repeat WBRT [76].

### Systemic therapies

While radiation therapy is the cornerstone of the treatment of SCLC brain metastases, the role of systemic therapies has long been under investigation (Table 2). While at this time there is no data to support the routine use of systemic therapies in newly diagnosed SCLC brain metastases [77, 78], this therapeutic approach has the potential to change our management paradigm in the not-to-distant future. A number of studies over the past few decades have evaluated systemic therapies in patients with both newly diagnosed and recurrent SCLC brain metastases [79–91]. All of the studies have utilized traditional cytotoxic chemotherapies and not targeted therapies as has been seen with other solid tumor brain metastases. These trials looked at chemotherapy alone or in conjunction with WBRT. Some studies have also included histologies other than SCLC. Direct comparison between these studies is difficult for a number of reasons which include the different eras in which they were conducted, different endpoints, and different study designs. To complicate the picture further numerous trials of systemic therapies for extensive-stage SCLC allowed for the presence of brain metastases, but not all have evaluated CNS-specific endpoints.

**Table 2:** Trials of systemic therapies for SCLC brain metastases

Author	Year	Phase	*n*	Intervention	OS (SCLC)	CNS RR (SCLC)
Chen	2012	2	36	WBRT (30 Gy) + etoposide + cisplatin	19.2 mo	76.5%
Liu	2010	3*	39	WBRT (36 Gy) followed by teniposide + cisplatin WBRT (36 Gy) with concomitant teniposide + cisplatin	NA	NA
Neuhaus	2009	3	96 total (33 SCLC)	WBRT (40 Gy) WBRT (40 Gy) with concomitant topotecan	NA	NA
Chen	2008	2	80 total (15 with SCLC BM)	Irinotecan + carboplatin	6 mo	NA
Lorusso	2006	2	19 total (3 SCLC)	topotecan	NA	66%
Omuro	2006	1	21 total (3 SCLC)	vinorelbine + temozolomide	NA	0%
Korfel	2002	2	30	topotecan	3.6 mo	33%
Postmus	2000	3	128	teniposide teniposide + WBRT (30 Gy)	3.2 mo 3.5 mo	22% 57%
Tummarrello	1998	NA	23 total (9 SCLC)	Cyclophosphamide + doxorubicin + vincristine + teniposide *or* cyclophosphamide + doxorubicin + vincristine followed by cisplatin + etoposide	NA	56%
Kaba	1997		115 total (9 SCLC)	TPDC-FuHu	NA	NA
Malacarne	1996		30 total (12 SCLC)	carboplatin + etoposide	23 weeks	NA
Postmus	1995	2	11	teniposide	NA	33%
Twelves	1990	3	610 total (19 SCLC)	cyclophosphamide + vincristine + etoposide	28 weeks	53%
Lee	1989		14	Cyclophosphamide + doxorubicin + vincristine + etoposide followed by WBRT	34 weeks	82%

OS, overall survival; CNS RR, central nervous system response rate; NA, not available; NSCLC, non-small-cell lung cancer; SCLC, small-cell lung cancer; Gy, grey; mo, months; BM, brain metastases; TPDC-FuHu, thioguanine, procarbazine, dibromodulcitol, CCNU, fluorouracil, hydroxyurea. *preliminary results.

The agents which have been investigated in this setting all have some degree of activity in extra-CNS SCLC. They fall into the broad categories of topoisomerase inhibitors (etoposide, teniposide, doxorubicin, topotecan, irinotecan), platinum agents (cisplatin, carboplatin), vinca alkaloids (vincristine, vinorelbine), and alkylating agents (cyclophosphamide, temozolomide). The role of immunotherapy (such as pembrolizumab or nivolumab with ipilumimab) is beginning to emerge for SCLC [92, 93]. However, immunotherapy has not been systematically studied in SCLC CNS metastases. CNS responses have been seen across a range of treatment regimens, supporting potential efficacy in the target organ with no clear signal for superiority of one regimen over another. However, despite CNS responses in a substantial number of patients, OS remains dismal across numerous studies.

The radiographic responses to systemic therapies are, however, heartening. They force us to push against the dogma that most systemically delivered agents are excluded from the nervous system. This needs to be considered on both a histology-specific and therapy-specific basis. Our current understanding of the ability of specific therapeutic agents to cross into the healthy and the diseased CNS is limited. This is in large part due to the practical limitation of performing pharmacokinetic (PK) studies on CNS tissue in living humans.

## CONCLUSIONS

The suboptimal outcomes which continue to be seen in patients with SCLC brain metastases warrant the need for further investigation. The robust radiographic response rates provide clear evidence for the biologic activity of our current treatment modalities. Their limited effect, however, on improving survival support the need for additional advances. Ongoing efforts to limit the toxicity of radiotherapy may prove beneficial in this patient population as well as in other solid tumors. The ability of systemically administered treatments, including those traditionally thought to have limited CNS penetration, holds out even greater hope. Conventional cytotoxic chemotherapy, targeted therapies/immunotherapies, and/or a combination of these have the potential to improve OS in this recalcitrant disease.

While there currently are no reliable prognostic or predictive molecular biomarkers in SCLC as our understanding of the disease evolves this will hopefully change. The study of SCLC brain metastases will continue to be limited by the paucity of tissue samples for this type of research. However, it should be noted that the neuropathologic work as well as imaging and therapeutic studies described above are beginning to shed light on how we may best address the problem of SCLC brain metastases. Refining our existing therapeutic modalities such as WBRT to limit their toxicity will be an impactful advance. In addition to the promise of hippocampal avoidance, further tailoring of the radiation fields informed by neuroimaging studies could improve efficacy while decreasing toxicity. Systemic therapies actively targeting SCLC brain metastases are also of interest. Potential targets include components of the angiogenic pathway such as SUR1 or a host of potential immunotherapeutic targets. Parceling out the answers to CNS-specific questions from large therapeutic trials which include patients with brain metastases may provide us with important insights and adequate safety and efficacy signals to justify moving forward with brain metastases-specific trials. Finally, the prevention of brain metastases in the SCLC patient population would be an important advance. The targeting of chemokines and adhesion molecules may play a role in achieving this goal. While improvements in survival for patients with SCLC brain metastases have been limited, the groundwork for important advances is present.

## References

[1] Chen LF, Patel JD, Lukas RV (2016). Advances in brain metastases presented at the American Society of Clinical Oncology 2016 Annual Meeting: part I. Future Oncol.

[2] Chen LF, Patel JD, Lukas RV (2016). Advances in brain metastases presented at the American Society of Clinical Oncology 2016 Annual Meeting: part II. Future Oncol.

[3] Lukas RV, Gabikian P, Garza M, Chmura SJ (2014). Treatment of brain metastases. Oncology.

[4] Lin X, DeAngelis LM (2015). Treatment of brain metastases. J Clin Oncol.

[5] Bertolini F, Spallanzani A, Fontana A, Depenni R, Luppi G (2015). Brain metastases: an overview. CNS Oncol.

[6] Dagogo-Jack I, Gill CM, Cahill DP, Santagata S, Brastianos PK (2017). Treatment of brain metastases in the modern genomic era. Pharmacol Ther.

[7] Dawe DE, Greenspoon JN, Ellis PM (2014). Brain metastases in non-small-cell lung cancer. Clin Lung Cancer.

[8] Lukas RV, Lesniak MS, Salgia R (2014). Brain metastases in non-small-cell lung cancer: better outcomes through current therapies and utilization of molecularly targeted approaches. CNS Oncol.

[9] Lukas RV, Kumthekar P, Rizvi S, Salgia R (2016). Systemic Therapies in the Treatment of non-small cell lung cancer brain metastases. Future Oncol.

[10] Bunn PA, Minna J, Augustyn A, Gazdar AF, Ouadah Y, Krasnow MA, Berns A, Brambilla E, Rekhtman N, Massion PP, Niederst M, Peifer M, Yokota J (2016). Small cell lung cancer: can recent advances in biology and molecular biology be translated into improved outcomes?. J Thorac Oncol.

[11] Goncalves PH, Peterson SL, Vigneau FD, Shore RD, Quarshie WO, Islam K, Schwartz AG, Wozniak AJ, Gadgeel SM (2016). Risk of brain metastases in patients with non-metastatic lung cancer: Analysis of the Metropolitan Detroit Surveillance, Epidemiology, and End Results (SEER) data. Cancer.

[12] Roengvoraphoj O, Eze C, Niyazi M, Li M, Hildebrandt G, Fietkau R, Belka C, Manapov F (2017). Prognostic role of patient gender in limited-disease small-cell lung cancer treated with chemo radiotherapy. Strahlenther Onkol.

[13] Gong L, Wang QI, Zhao L, Yuan Z, Li R, Wang P (2013). Factors affecting the risk of brain metastasis in small cell lung cancer with surgery: is prophylactic cranial irradiation necessary for stage I–III disease?. Int J Radiat Oncol Biol Phys.

[14] Seute T, Leffers P, ten Velde GP, Twijnstra A (2005). Leptomeningeal metastases from small cell lung carcinoma. Cancer.

[15] Rosen ST, Aisner J, Makuch RW, Matthews MJ, Ihde DC, Whitacre M, Glatstein EJ, Wiernik PH, Lichter AS, Bunn PA (1982). Carcinomatous leptomeningitis in small cell lung cancer: a clinicopathologic review of the National Cancer Institute experience. Medicine.

[16] Videtic GM, Adelstein DJ, Mekhail TM, Rice TW, Stevens GH, Lee SY, Suh JH (2007). Validation of the RTOG recursive partitioning analysis (RPA) classification for small-cell lung cancer-only brain metastases. Int J Radiat Oncol Biol Phys.

[17] Sperduto PW, Kased N, Roberge D, Xu Z, Shanley R, Luo X, Sneed PK, Chao ST, Weil RJ, Suh J, Bhatt A, Jensen AW, Brown PD (2012). Summary report on the graded prognostic assessment: an accurate and facile diagnosis-specific tool to estimate survival for patients with brain metastases. J Clin Oncol.

[18] Rades D, Dziggel L, Segedin B, Oblak I, Nagy V, Marita A, Schild SE (2013). The first survival score for patients with brain metastases from small cell lung cancer (SCLC). Clin Neurol Neurosurg.

[19] Piaget S (1889). The distribution of secondary growths in cancer of the breast. Lancet.

[20] Brastianos PK, Carter SL, Santagata S, Cahill DP, Taylor-Weiner A, Jones RT, Van Allen EM, Lawrence MS, Horowitz PM, Cibulskis K, Ligon KL, Tabernero J, Seoane J (2015). Genomic characterization of brain metastases reveals branched evolution and potential therapeutic targets. Cancer Discov.

[21] Bender ET, Tome WA (2011). Distribution of brain metastases: implications for non-uniform dose prescriptions. Br J Radiol.

[22] Graf AH, Buchberger W, Langmayr H, Schmid KW (1988). Site preference of metastatic tumours of the brain. Virchows Arch A Pathol Anat Histopathol.

[23] Quattrocchi CC, Errante Y, Gaudino C, Mallio CA, Giona A, Santini D, Tonini G, Zobel BB (2012). Spatial brain distribution of intra-axial metastatic lesions in breast and lung cancer patients. J Neurooncol.

[24] Takano K, Kinoshita M, Takagaki M, Sakai M, Tateishi S, Achiha T, Hirayama R, Nishino K, Uchida A, Kumagai T, Okami J, Kawaguchi A, Hashimoto N (2016). Different spatial distributions of brain metastases from lung cancer by histological subtype and mutation status of epidermal growth factor receptor. Neuro Oncol.

[25] Berghoff AS, Rajky O, Winkler F, Bartsch R, Furtner J, Hainfellner JA, Goodman SL, Weller M, Schittenhelm J, Preusser M (2013). Invasion patterns in brain metastases of solid cancers. Neuro Oncol.

[26] Sculier JP, Feld R, Evans WK, DeBoer G, Shepherd FA, Payne DG, Pringle JF, Yeoh JL, Quirt IC, Curtis JE, Myers R, Herman JG (1987). Neurologic disorders in patients with small cell lung cancer. Cancer.

[27] Ilhan-Mutlu A, Siehs C, Berghoff AS, Ricken G, Widhalm G, Wagner L, Preusser M (2016). Expression-profiling of angiogenesis related genes in brain metastases of lung cancer and melanoma. Tumor Biol.

[28] Thompson EM, Pischko GL, Muldoon LL, Neuwelt EA (2013). Inhibition of SUR1 decreases the vascular permeability of cerebral metastases. Neoplasia.

[29] Kafka A, Tomas D, Beros V, Pecina HI, Zeljko M, Pecina-Slaus N (2014). Brain metastases from lung cancer show increased expression of DVL1, DVL3, and beta-catenin and down-regulation of E-cadherin. Int J Mol Sci.

[30] Mauri FA, Pinato DJ, Trivedi P, Sharma R, Shiner RJ (2012). Isogeneic comparison of primary and metastatic lung cancer identifies CX3CR1 as a molecular determinant of site-specific metastatic diffusion. Oncol Rep.

[31] Kuo CS, Krasnow MA (2015). Formation of neurosensory organ by epithelial cell slithering. Cell.

[32] Reaux-Le Goazigo A, Van Steenwinkle J, Rostene W, Melik Parsadaniantz S (2013). Current status of chemokines in the adult CNS. Prog Neurobiol.

[33] Gangadhar T, Nandi S, Salgia R (2010). The role of chemokine receptor CXCR4 in lung cancer. Cancer Biol Ther.

[34] Tabaka J, Nowacki P, Pankowski J (2006). The interaction between lung cancer metastases to the brain and their surroundings. Folia Neuropathol.

[35] Chen Q, Boire A, Jin X, Valiente M, Er EE, Lopez-Soto A, Jacob LS, Patwa R, Shah H, Xu K, Cross JR, Massague J (2016). Carcinoma-astrocyte gap junctions promote brain metastases by cGAMP transfer. Nature.

[36] Berghoff AS, Ricken G, Wilhem D, Rajky O, Widhalm G, Dieckmann K, Birner P, Bartsch R, Preusser M (2016). Tumor infiltrating lymphocytes and PD-L1 expression in brain metastases of small cell lung cancer (SCLC). J Neurooncol.

[37] Yan F, Pang J, Peng Y, Molina JR, Yang P, Liu S (2016). Elevated cellular PD1/PD-L1 expression confers acquired resistance to cisplatin in small cell lung cancer cells. PLoS One.

[38] George J, Lim JS, Jang SJ, Cun Y, Ozretic L, Kong G, Leenders F, Lu X, Fernandez-Cuesta L, Bosco G, Mueller C, Dahmen I, Jahchan NS (2015). Comprehensive genomic profiles of small cell lung cancer. Nature.

[39] Sequist LV, Waltmann BA, Dias-Santagata D, Digumarthy S, Turke AB, Fidias P, Bergethon K, Shaw AT, Gettinger S, Cosper AK, Akhavanfard S, Heist RS, Temel J (2011). Genotypic and histologic evolution of lung cancers acquiring resistance to EGFR inhibitors. Sci Transl Med.

[40] Jett JR, Schild SE, Kesler KA, Kalemkerian GP

[41] Rudin CM, Ismaila N, Hann CL, Malhotra N, Movsas B, Norris K, Pietanza MC, Ramalingam SS, Turrisi AT, Giaccone G (2015). Treatment of Small-Cell Lung Cancer: American Society of Clinical Oncology Endorsement of the American College of Chest Physicians Guideline. J Clin Oncol.

[42] NCCN Clinical Practice Guidelines in Oncology Small Cell Lung Cancer. https://www.nccn.org/professionals/physician_gls/pdf/sclc.pdf.

[43] Nomoto Y, Miyamoto T, Yamaguchi Y (1994). Brain metastasis of small cell lung carcinoma: comparison of Gd-DTPA enhanced magnetic resonance imaging and enhanced computerized tomography. Jpn J Clin Oncol.

[44] Seute T, Leffers P, Wilmink JT, ten Velde GP, Twijnstra A (2006). Response of asymptomatic brain metastases from small-cell lung cancer to systemic first-line chemotherapy. J Clin Oncol.

[45] Seute T, Leffers P, ten Velde GP, Twijnstra A (2008). Detection of brain metastases from small cell lung cancer: consequences of changing imaging techniques (CT versus MRI). Cancer.

[46] Hayashida Y, Hirai T, Morishita S, Kitajima M, Murakami R, Korogi Y, Makino K, Nakamura H, Ikushima I, Yamura M, Kochi M, Kuratsu JI, Yamashita Y (2006). Diffusion-weighted imaging of metastatic brain tumors: comparison with histologic type and tumor cellularity. AJNR Am J Neuroradiol.

[47] Zakaria R, Das K, Radon M, Bhojak M, Rudland PR, Sluming V, Jenkinson MD (2014). Diffusion-weighted MRI characteristics of the cerebral metastasis to brain boundary predicts patient outcomes. BMC Med Imaging.

[48] Duygulu G, Ovali GY, Calli C, Kittis O, Yunten N, Akalin T, Islekel S (2010). Intracerebral metastasis showing restricted diffusion: correlation with histopathologic findings. Eur J Radiol.

[49] Meyer HJ, Fiedler E, Kornhuber M, Spielmann RP, Surov A (2015). Comparison of diffusion-weighted imaging findings in brain metastases of different origin. Clin Imaging.

[50] Bradley JD, Dehdashti F, Mintun MA, Govindan R, Trinkaus K, Siegel BA (2004). Positron emission tomography in limited-stage small-cell lung cancer: a prospective study. J Clin Oncol.

[51] Brink I, Schumacher T, Mix M, Rhuland S, Stoelben E, Digel W, Henke M, Ghanem N, Moser E, Nitzsche EU (2004). Impact of [18F]FDG-PET on the primary staging of small-cell lung cancer. Eur J Nucl Med Mol Imaging.

[52] Lee HY, Chung JK, Jeong JM, Lee DS, Kim DG, Jung HW, Lee MC (2008). Comparison of FDG-PET findings of brain metastasis from non-small-cell lung cancer and small-cell lung cancer. Ann Nucl Med.

[53] Hochstenbag MM, Heidendal GA, Wouters EF, ten Velde GP (1997). In-111 octreotide imaging in staging of small cell lung cancer. Clin Nucl Med.

[54] Sollini M, Farioli D, Froio A, Chella A, Asti M, Boni R, Grassi E, Roncali M, Versari A, Erba PA (2013). Brief report on the use of radiolabeled somatostatin analogs for the diagnosis and treatment of metastatic small-cell lung cancer patients. J Thorac Oncol.

[55] Glaudemans AW, Enting RH, Heesters MA, Dierskx RA, van Rheenen RW, Walenkamp AM, Slart RH (2013). Value of 11C-methionine PET in imaging brain tumours and metastases. Eur J Nucl Med Mol Imaging.

[56] Juhasz C, Dwivedi S, Kamson DO, Michelhaugh SK, Mittal S (2014). Comparison of amino acid positron emission tomographic radiotracers for molecular imaging of primary and metastatic brain tumors. Mol Imaging.

[57] Weber WA, Wester HJ, Grosu AL, Herz M, Dzewas B, Feldman HJ, Molls M, Stocklin G, Schwaiger M (2000). O-(2-[18F]fluoroethyl)-L-tyrosine and L-[methyl-11C]methionine uptake in brain tumours: initial results of a comparative study. Eur J Nucl Med.

[58] Terakawa Y, Tsuyuguchi N, Iwai Y, Yamanak K, Higashiyama S, Takami T, Ohata K (2008). Diagnostic accuracy of 11C-methionine PET for differentiation of recurrent brain tumors from radiation necrosis after radiotherapy. J Nucl Med.

[59] Tsuyuguchi N, Sunada I, Iwai Y, Yamanaka K, Tanaka K, Takami T, Otsuka Y, Sakamoto S, Ohata K, Goto T, Hara M (2003). Methionine positron emission tomography of recurrent metastatic brain tumor and radiation necrosis after stereotactic radiosurgery: is a differential diagnosis possible?. J Neurosurg.

[60] Kamson DO, Mitta S, Buth A, Muzik O, Kupsky WJ, Robinette NL, Barger GR, Juhasz C (2013). Differentiation of glioblastomas from metastatic brain tumors by tryptophan uptake and kinetic analysis: a positron emission tomographic study with magnetic resonance imaging comparison. Mol Imaging.

[61] Patchell RA, Tibbs PA, Walsh JW, Dempsey RJ, Maruyama Y, Kryscio RJ, Markesbery WR, Macdonald JS, Young B (1990). A randomized trial of surgery in the treatment of single metastases to the brain. N Engl J Med.

[62] Vecht CJ, Haaxma-Reiche H, Noordijk EM, Padberg GW, Voormolen JH, Hoekstra FH, Tans JT, Lambooij N, Metsaars JA, Wattendorf AR, Brand R, Hermans J (1993). Treatment of single brain metastasis: radiotherapy alone or combined with surgery. Ann Neurol.

[63] Mintz AH, Kestle J, Rathbone MP, Gaspar L, Hugenholtz H, Fisher B, Duncan G, Skingley P, Foster G, Levine M (1996). A randomized trial to assess the efficacy of surgery in addition to radiotherapy in patients with a single cerebral metastasis. Cancer.

[64] Hochstenbag MM, Twinjstra A, Wilmink JT, Woters EF, ten Velde GP (2000). Asymptomatic brain metastases (BM) in small cell lung cancer (SCLC): MR-imaging is useful at initial diagnosis. J Neurooncol.

[65] Auperin A, Arriagada R, Pignon JP (1999). Prophylactic cranial irradiation for patients with small-cell lung cancer in complete remission. Prophylactic Cranial Irradiation Overview Collaborative Group. N Engl J Med.

[66] Slotman B, Faivre-Finn C, Kramer G, Rankin E, Snee M, Hatton M, Postmus PE, Collette L, Musat E, Senan S (2007). Prophylactic cranial irradiation in extensive small-cell lung cancer. N Engl J Med.

[67] Lok BH, Pietanza MC, Foster A, Rudin CM, Perez CA, Ong L, Krug L, Rimner A, Wu AJ (2015). The factors influencing the utilization of prophylactic cranial irradiation in limited-stage small cell lung cancer. Int J Radiat Oncol Biol Phys.

[68] Wolfson AH, Bae K, Komaki R, Meyers C, Movsas B, Le Pechoux C, Werner-Wasik M, Videtic GM, Garces YI, Choy H (2011). Primary analysis of a phase II randomized trial Radiation Therapy Oncology Group (RTOG) 0212: Impact of different total doses and schedules of prophylactic cranial irradiation on chronic neurotoxicity and quality of life for patients with limited-disease small-cell lung cancer. Int J Radiat Oncol Biol Phys.

[69] Gondi V, Paulus R, Bruner DW, Meyers CA, Gore EM, Wolfson A, Werner-Wasik M, Sun AY, Choy H, Movsas B (2013). Decline in tested and self-reported cognitive functioning after prophylactic cranial irradiation for lung cancer: Pooled secondary analysis of radiation therapy oncology group randomized trials 0212 and 0214. Int J Radiat Oncol Biol Phys.

[70] Sun A, Bae K, Gore EM, Movsas B, Wong SJ, Meyers CA, Bonner JA, Schild SE, Gaspar LE, Bogart JA, Werner-Wasik M, Choy H (2011). Phase III trial of prophylactic cranial irradiation compared with observation in patients with locally advanced non-small-cell lung cancer: Neurocognitive and quality-of-life analysis. J Clin Oncol.

[71] Li J, Bentzen S, Li J, Renschler M, Mehta MP (2007). Regression after whole-brain radiation therapy for brain metastases correlates with survival and improved neurocognitive function. J Clin Oncol.

[72] Brown PD, Jaeckle K, Ballman KV, Farace E, Cerhan JH, Anderson SK, Carrero XW, Barker FG, Deming R, Burri SH, Menard C, Chung C, Stieber VW (2016). Effect of radiosurgery alone versus radiosurgery with whole-brain radiation therapy on cognitive function in patients with 1 to 3 brain metastases: A randomized clinical trial. JAMA.

[73] Gondi V, Tome WA, Mehta MP (2010). Why avoid the hippocampus? A comprehensive review. Radiather Oncol.

[74] Gondi V, Pugh SL, Tome WA, Caine C, Corn B, Kanner A, Rowley H, Kundapur V, DeNittis A, Greenspoon JN, Konski AA, Bauman GS, Shah S (2014). Preservation of memory with conformal avoidance of the hippocampal neural stem cell compartment during whole-brain radiotherapy for brain metastases (RTOG 0933): A phase 2 multi-institutional trial. J Clin Oncol.

[75] Kundapur V, Ellchuk T, Ahmed S, Gondi V (2015). Risk of hippocampal metastases in small cell lung cancer at presentation and after cranial irradiation: A safety profile study for hippocampal sparing during prophylactic or therapeutic cranial irradiation. Int J Radiat Oncol Biol Phys.

[76] Wegner RE, Olson AC, Kondziolka D, Niranjan A, Lundsford LD, Flickinger JC (2011). Stereotactic radiosurgery for patients with brain metastasis from small cell lung cancer. Int J Radiat Oncol Biol Phys.

[77] Mehta MP, Paleologos NA, Mikkelsen T, Robinson PD, Ammirati M, Andrews DW, Asher AL, Burri SH, Cobbs CS, Gaspar LE, Kondziolka D, Linskey ME, Loeffler JS (2010). The role of chemotherapy in the management of newly diagnosed brain metastases: a systematic review and evidence-based clinical practice guideline. J Neurooncol.

[78] Reveiz L, Rueda JR, Cardona AF (2012). Chemotherapy for brain metastases from small cell lung cancer. Cochrane Database Syst Rev.

[79] Chen LK, Huang H, Liao H, Liu GZ, Zeng YD, Dinglin XX, Xu GC, Wei WD (2012). Chemotherapy with concurrent brain and thoracic radiotherapy in brain-only metastases of treatment naïve small-cell lung cancer: a phase II study. Med Oncol.

[80] Liu M, Zhou Y, Han Q, Gao T, Luo Z, Wang W (2010). Whole brain radiotherapy concomitant or sequential Vm26/DDP in treating small cell lung cancer patients with brain metastases. Chinese-German J Clin Oncol.

[81] Neuhaus T, Ko Y, Muller RP, Grabenbauer GG, Hedde JP, Schueller H, Kocher M, Stier S, Fietkau R (2009). A phase III trial of topotecan and whole brain radiation therapy for patients with CNS-metastases due to lung cancer. Br J Cancer.

[82] Chen G, Huynh M, Chen A, Fehrenbacher L, Gandara D, Lau D (2008). Chemotherapy for brain metastases in small-cell lung cancer. Clin Lung Cancer.

[83] Lorusso V, Galetta D, Giotta DF, Rinaldi A, Romito S, Brunetti C, Silvestris N, Colucci G (2006). Topotecan in the treatment of brain metastases: A phase II study of GOIM (Gruppo Oncologico dell’Ittalia Meridionale). Anticancer Res.

[84] Omuro AM, Raizer JJ, Demopoulos A, Malkin MG, Abrey LE (2006). Vinorelbine combined with a protracted course of temozlomide for recurrent brain metastases: a phase I trial. J Neurooncol.

[85] Korfel A, Oehm C, von Pawel J, Keppler U, Depperman M, Kaubitsch S, Thiel E (2002). Response to topotecan of symptomatic brain metastases of small-cell lung cancer also after whole-brain irradiation: a multicenter phase II study. Eur J Cancer.

[86] Postmus PE, Haaxma-Reiche H, Smit EF, Groen HJ, Karnicka H, Lewinski T, van Meerbeeck J, Clerico M, Gregor A, Curran D, Sahmoud T, Kirkpatrick A, Giaccone G (2000). Treatment of brain metastases of small-cell lung cancer: comparing teniposide and teniposide with whole-brain radiotherapy-a phase III study of the European Organization for the Research and Treatment of Cancer Lung Cancer Cooperative Group. J Clin Oncol.

[87] Tummarello D, Lippe P, Bracci R, Mari D, Monterubianessi MC, Battelli N (1998). First line chemotherapy in patients with brain metastases from non-small and small cell lung cancer. Oncol Report.

[88] Kaba SE, Kyritsis AP, Hess K, Yung WK, Mercier R, Dakhil S, Jaeckle KA, Levin VA (1997). TPDC-FuHu chemotherapy for the treatment of recurrent metastatic brain tumors. J Clin Oncol.

[89] Malacarne P, Santini A, Maestri A (1996). Response of brain metastases from lung cancer to systemic chemotherapy to carboplatin and etoposide. Oncology.

[90] Postmus PE, Smit EF, Haaxma-Reiche H, van Zandwijk N, Ardizzoni A, Quoix E, Kirkpatrick A, Sahmoud T, Giaccone G (1995). Teniposide for brain metastases of small-cell lung cancer: a phase II study. European Organization for Research and Treatment of Cancer Lung Cancer Cooperative Group. J Clin Oncol.

[91] Twelves CJ, Souhami RL, Harper PG, Ash CM, Spiro SG, Earl HM, Tobias JS, Quinn H, Geddes DM (1990). The response of cerebral metastases in small cell lung cancer to systemic chemotherapy. Br J Cancer.

[92] Horn L, Reck M, Spigel DR (2016). The future of immunotherapy in the treatment of small cell lung cancer. Oncologist.

[93] Paglialunga L, Salih Z, Ricciuti B, Califano R (2016). Immune checkpoint blockade in small cell lung cancer: is there a light at the end of the tunnel?. ESMO Open.

